# Clinical characteristics, treatment, and prognosis of 118 cases of myeloid sarcoma

**DOI:** 10.1038/s41598-022-10831-7

**Published:** 2022-04-26

**Authors:** Haiqiu Zhao, Zhenkun Dong, Dingming Wan, Weijie Cao, Haizhou Xing, Zhenzhen Liu, Jixin Fan, Haiqiong Wang, Runqing Lu, Yinyin Zhang, Qianqian Cheng, Zhongxing Jiang, Fei He, Xinsheng Xie, Rong Guo

**Affiliations:** 1 Department of Hematology, The First Affiliated Hospital of Zhengzhou University, No. 1 Jianshe East Road, Zhengzhou, 450052 Henan China; 2 State Key Laboratory of Experimental Hematology, Institute of Hematology and Blood Diseases Hospital, Chinese Academy of Medical Sciences and Peking Union Medical College, Tianjin, 300020 China; 3 Department of Cardiology, The First Affiliated Hospital of Zhengzhou University, Zhengzhou, 450052 Henan China

**Keywords:** Cancer, Haematological diseases

## Abstract

Myeloid sarcoma is a rare manifestation of acute myeloid leukemia (AML) and is associated with poor overall survival (OS). The optimal treatment remains unclear. The study retrospectively evaluated 118 patients with myeloid sarcoma who were treated at the First Affiliated Hospital of Zhengzhou University from January 2010 to July 2021. All cases were diagnosed by tissue biopsy. 41 patients underwent genetic mutation analysis. The most frequent genetic mutations were *KIT* (16.6%), followed by *TET2* (14.6%), and *NRAS* (14.6%). The median survival time of 118 patients was 4 months (range, 1–51 months), while the median survival time of 11 patients who received allogeneic hematopoietic stem cell transplantation (allo-HSCT) was 19 months (range, 8–51 months). 4 (36.4%) of the 11 patients experienced relapse within 1 year after transplantation. 1 patient died from a severe infection. Of the 6 surviving patients, 5 patients have received maintenance treatment with decitabine after transplantation, and all remained in a state of recurrence-free survival. Patients with myeloid sarcoma have a very unfavorable outcome. Allo-HSCT is an effective treatment option. Recurrence remains the main cause of transplant failure. Maintenance treatment with decitabine after transplantation can prolong the recurrence-free survival time, although these results must be verified in a study with expanded sample size.

## Introduction

Myeloid sarcoma is a rare extramedullary tumor formed by the proliferation of immature myeloid precursor cells. The World Health Organization (WHO) defines it as a unique type of acute myeloid leukemia (AML) with various clinical manifestations^1,2^. Myeloid sarcoma can manifest as primary myeloid sarcoma without bone marrow involvement, myeloid sarcoma with peripheral blood or bone marrow involvement, extramedullary recurrence after AML remission, or the progressive form of myelodysplastic syndrome (MDS), myeloproliferative neoplasm (MPN), and chronic myeloid leukemia (CML). Because of the low incidence of myeloid sarcoma, most descriptions of the disease are case reports and retrospective analyses with small sample sizes. The prognosis of myeloid sarcoma is poor and the survival time is short. Clarification of the relevant risk factors that affect the prognosis of myeloid sarcoma will guide clinical stratified treatment and prolong overall survival (OS). Allogeneic hematopoietic stem cell transplantation (allo-HSCT) can be used as a first-line regimen for consolidation therapy after induced remission and as a salvage scheme for patients with relapsed and refractory disease^3^. However, recurrence remains a major cause of transplant failure, and the recurrence rate is 50% after transplantation in myeloid sarcoma^4^. Extramedullary relapse as a solitary event precedes hematologic relapse, occurs frequently, and remains a clinically challenging situation with few therapeutic options^5^. Extramedullary tissues have reduced immunologic surveillance that facilitates cancer immune evasion. This mechanism of immune escape is also involved in extramedullary AML relapse after allo-HSCT; studies have found that graft-versus-leukemia (GVL) reactions are less effective in tissues with reduced immunologic surveillance, in which the numbers of patrolling donor T and natural killer (NK) cells are limited^6,7^. Decitabine can enhance the expression of NK cells and cytotoxic CD8^+^ T cells^8^, it can increase the immune recognition of donor-derived T cells to leukemic cells after transplantation. The main role of demethylation maintenance therapy after allo-HSCT is to prevent leukemia recurrence and reduce graft-versus-host disease (GVHD)^9–11^. In this study, 118 patients with myeloid sarcoma were enrolled to analyze their clinical characteristics and the effect of different treatment methods on prognosis. Among them, 11 patients with myeloid sarcoma received allo-HSCT, and 6 patients received low-dose decitabine maintenance therapy after transplantation. So far, 5 patients have survived without recurrence.

## Results

### Patient characteristics

The general characteristics of the patients in this study are shown in Table 1. This study included 118 patients with myeloid sarcoma: 48 (40.7%) had primary myeloid sarcoma, 51 (43.2%) had concurrent intramedullary lesions, and 19 (16.1%) had relapsed myeloid sarcoma. The most common intramedullary lesions were AML (40/51), followed by MDS (9/51) and CML (2/51). In general, 73 men (61.9%) and 45 women (38.1%) were enrolled. The male-to-female ratio was 1.6 to 1, and the median age of onset was 44 years (range, 1–81 years). There was a total of 141 involved sites of myeloid sarcoma (Table 2). The most common sites were lymph nodes (22.7%), followed by soft tissue (14.2%), the spinal canal (10.6%), the digestive tract (6.4%), and the genital system (6.4%). Twenty patients (16.9%) had more than two sites involved. Sex, age of onset, and single or multiple sites of involvement had no statistical significance on the prognosis of myeloid sarcoma (*P* > 0.05).

**Table 1:** Patients and characteristics in the diagnosis of MS.

Characteristics	Data
**Sex**	
Male/female	73/45
Median age (range), years	44 (1–81)
**MS subtype**	
Primary MS	48
MS with intramedullary disease	51
Relapsed MS	19
**No. of extramedullary sites involved**	
Single site	98
≥ 2	20
**Karyotype (no./available)**	
Normal	22/39
t(8;21) and inv(16)	10/39
Complex	2/39
Other^a^	5/39
**IHC (no./available)**	
MPO	94/110
CD43	54/54
CD117	38/61
CD68	13/21
CD56	11/26
Median Ki-67 index, % (range)	60 (10–95)
**Treatment**	
Local treatment	31/118
Chemotherapy	76/118
Transplantation	11/118

*MS* myeloid sarcoma, *IHC* immunohistochemistry; ^a^t(9;22), t(6;11), t(9;11), der(7;8), 5q^−^.

**Table 2:** The involved sites of MS in 118 patients.

Involved site	Primary MS	MS with intramedullary disease	Relapsed MS	All patients
Lymph nodes	11	19	2	32
Soft tissues	5	11	4	20
Spinal canal	8	6	1	15
Digestive tract	4	5	0	9
Genital system	6	2	1	9
Pleura	3	4	1	8
Skin	1	1	5	7
Nasopharynx	3	4	0	7
Lung	5	0	0	5
Bone	3	0	2	5
Mediastinum	1	2	1	4
Brain	1	0	3	4
Breast	2	1	1	4
Orbit	1	2	0	3
Gingiva	2	1	0	3
Parotid	3	0	0	3
Other^a^	0	2	1	3

*MS* myeloid sarcoma. ^a^Optic nerve, bladder, spleen.

### Immunohistochemistry

The positive expression rates of myeloperoxidase, CD117, and CD68 were 85.5% (94/110), 62.3% (38/61), and 61.9% (13/21), respectively. In addition, 100% and 42.3% of cases expressed CD43 and CD56, respectively. The median Ki-67 index was 60% (range, 10–95%). The survival analysis of 26 patients with the expression of CD56 showed that CD56 was not a significant variable affecting the prognosis of myeloid sarcoma (*P* = 0.30).

### Karyotype and molecular genetics

Of the118 patients, 39 underwent karyotyping analysis; 22 (56.4%) had a normal karyotype and 17 (43.6%) had an abnormal karyotype. Among the abnormal karyotypes, nine were t(8;21) translocations, one was inv(16), and two were complex karyotypes. The survival analysis stratified by chromosome karyotypes showed that there was no significant influence on the prognosis of myeloid sarcoma based on an abnormal karyotype (*P* = 0.93).

**Figure 1:**
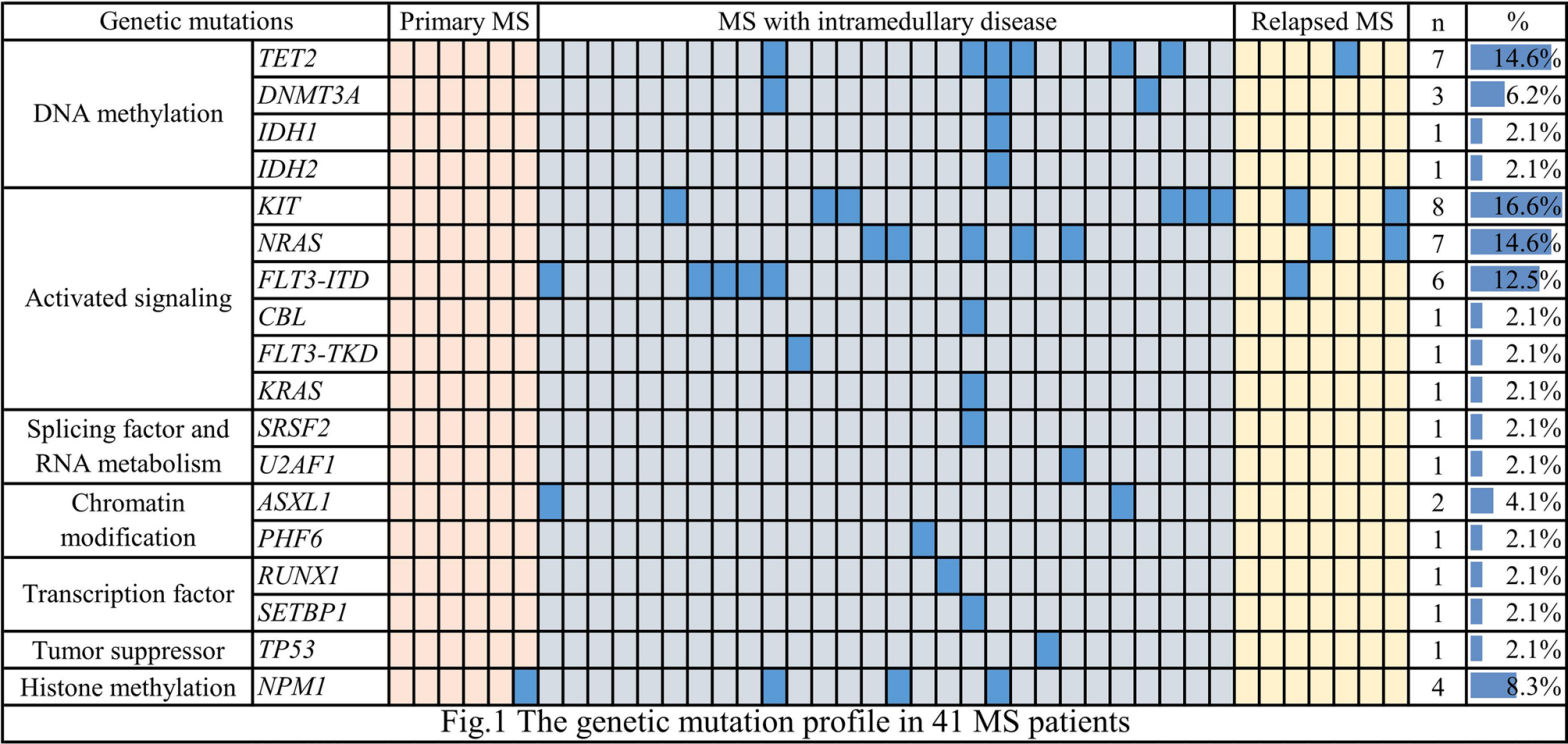
The genetic mutation profile in 41 myeloid sarcoma patients.

Forty-one patients underwent genetic mutation analysis (Fig. 1). The most frequent genetic mutations were *KIT* (16.6%), followed by *TET2* (14.6%), *NRAS* (14.6%), *FLT3-ITD* (12.5%), *NPM1* (8.3%), and *DNMT3A* (6.2%).

### Treatment and outcome

In the primary myeloid sarcoma group, 25 patients received local treatment, and 23 patients received chemotherapy. Among patients with intramedullary disease, 5 patients received local treatment, 37 patients received chemotherapy, and 9 patients received allo-HSCT. In the relapsed myeloid sarcoma group, 1 patient received local treatment, 16 patients received chemotherapy, and 2 patients received allo-HSCT.

The total number of patients with relapsed myeloid sarcoma after AML treatment in this study was 19, of which 8 patients received transplants for their primary AML before the myeloid sarcoma, and no patients received transplants again after the myeloid sarcoma. The details of the 8 patients who relapsed after transplantation are shown in Table 3. Of the 19 patients with relapsed myeloid sarcoma, 8 (42.1%) had concurrent intramedullary relapse, and 11 (57.9%) had only extramedullary relapse (no bone marrow involvement). During the follow-up period, 3 patients with pure extramedullary relapse were found to progress to bone marrow recurrence during chemotherapy, and the progression time was 3, 6, and 15 months after the diagnosis, respectively.

**Table 3:** Data of 8 cases with relapsed MS after transplantation.

Transplantation characteristics	N (%)
**Sex**	
Male/female	6/2
Median age at transplantation, years	21 (range, 11–38)
**Prior therapy**	
Chemotherapy	6 (75.0%)
Local treatment + chemotherapy	2 (25.0%)
**Disease status at HSCT**	
CR1	4 (50.0%)
CR2	2 (25.0%)
Refractory	1 (12.5%)
Relapsed	1 (12.5%)
**Stem-cell source**	
PBSC	6 (75.0%)
BM + PBSC	2 (25.0%)
Donor	
MSD	5 (62.5%)
MUD	2 (25.0%)
HID	1 (12.5%)
**Type of conditioning regimen**	
MAC	5 (62.5%)
RIC	3 (37.5%)
Time to ANC ≥ 0.5 × 10^9^/L, days	17 (range, 13–19)
**Acute GVHD, grade**	
I	3 (37.5%)
II–IV	0 (0)
**Chronic GVHD**	
Limited	1 (12.5%)
Extensive	0 (0)
Median time to relapse after HSCT, months	21 (range, 3–38)

*MS* myeloid sarcoma, *HSCT* hematopoietic stem cell transplantation, *CR1* first complete remission, *CR2* second complete remission, *PBSC* peripheral blood stem cell, *BM* bone marrow, *MSD* matched sibling donor, *MUD* matched unrelated donor, *HID* haploidentical donor, *MAC* myeloablative conditioning, *RIC* reduced-intensity conditioning, *ANC* absolute neutrophil count, *GVHD* graft-versus-host disease.

Of the 31 patients treated with local therapy, 1 patient survived, 29 died, and 1 was lost to follow-up; the median survival time was 1 month (range, 1–18 months). Among the 76 chemotherapy-treated patients, 9 survived, 55 died, and 12 were lost to follow-up, with a median survival time of 7 months (range, 1–48 months). Of the 11 patients in the allo-HSCT group, 6 survived, and 5 died, with a median survival time of 19 months (range, 8–51 months). Survival analysis according to different treatment regimens suggested that allo-HSCT offered a good prognosis (*P* < 0.001).

For the chemotherapy-treated group, these patients all received consolidation therapy after the induction regimen. Considering that there is a high possibility of recurrence after remission with extramedullary infiltration, the patients who are generally tolerated regularly receive consolidation chemotherapy containing high-dose cytarabine, for elderly or unfit patients receive demethylation therapy such as decitabine to control disease progression. Six patients with *FLT3-ITD* mutation received sorafenib-targeted therapy.

In this study, 11 patients in the MS with intramedullary disease group had previously been diagnosed with myeloid diseases, including 9 patients with MDS and 2 patients with CML. 81.8% patients (9/11) received corresponding treatment before progression to MS, 7 patients with MDS received treatment with demethylating drugs and 2 patients with CML received TKI therapy. Two MDS patients refused chemotherapy and only received local excision.

Data from 11 patients who received allo-HSCT are shown in Table 4. The male-to-female ratio was 1.7 to 1, and the median age was 22 years (range, 13–50 years). Eight patients had myeloid sarcoma with AML, one had myeloid sarcoma with MDS, and two had relapsed myeloid sarcoma. All patients received 7 + 3 standard induction chemotherapy before transplantation, followed by regular consolidation chemotherapy similar to the AML regimen. All 11 patients achieved minimal residual disease (MRD) negative CR before transplantation. Ten (90.9%) of the 11 patients received myeloablative conditioning (MAC) and underwent successful grafts within 20 days after transplantation, whereas 9.1% (1/11) of the patients received reduced-intensity conditioning (RIC) and died from a severe infection after graft failure. At the end of follow-up, 5 patients died, 6 patients were alive. 4 deaths were due to recurrence, and 1 death was due to infection. Of the 6 surviving patients, 5 patients have received maintenance therapy with decitabine after transplantation, and all remained in a state of recurrence-free survival.

**Table 4:** Characteristics of 11 MS patients who received HSCT.

No.	Age (years)	Sex (M/F)	Diagnosis	Site	Disease status	Conditioning intensity	Donor	Engraftment	GVHD	Cycles of decitabine	OS (months)	Outcome
013	30	M	MS with AML	Lymph nodes	CR1	MAC	MSD	Day 18	Chronic GVHD	8	51	Alive
033	50	M	MS with AML	Lymph nodes	CR1	MAC	MUD	Day 17	Acute GVHD	None	26	Dead
034	22	F	MS with AML	Orbit	CR1	MAC	MSD	Day 18	None	8	40	Alive
035	46	M	MS with MDS	Lymph nodes	CR1	MAC	MUD	Day 15	None	None	16	Dead
036	20	F	MS with AML	Breast	CR1	MAC	MSD	Day 19	None	None	8	Dead
039	19	F	MS with AML	Soft tissue	CR1	MAC	MSD	Day 20	None	None	15	Alive
073	21	F	Relapsed MS	Bone	CR1	MAC	MSD	Day 17	None	3	21	Dead
089	35	M	Relapsed MS	Skin	CR1	MAC	MSD	Day 15	None	6	19	Alive
094	42	M	MS with AML	Mediastinum	CR1	RIC	MSD	None	None	None	8	Dead
109	17	M	MS with AML	Lymph nodes	CR1	MAC	HID	Day 18	None	8	23	Alive
118	13	M	MS with AML	Spinal canal	CR1	MAC	MSD	Day 19	None	3	9	Alive

*MS* myeloid sarcoma, *AML* acute myeloid leukemia, *MDS* myelodysplastic syndrome, *HSCT* hematopoietic stem cell transplantation, *GVHD* graft-versus-host disease, *MAC* myeloablative conditioning, *RIC* reduced-intensity conditioning, *MSD* matched sibling donor, *MUD* matched unrelated donor, *HID* haploidentical donor, *OS* overall survival.

### Prognostic factors

**Figure 2:**
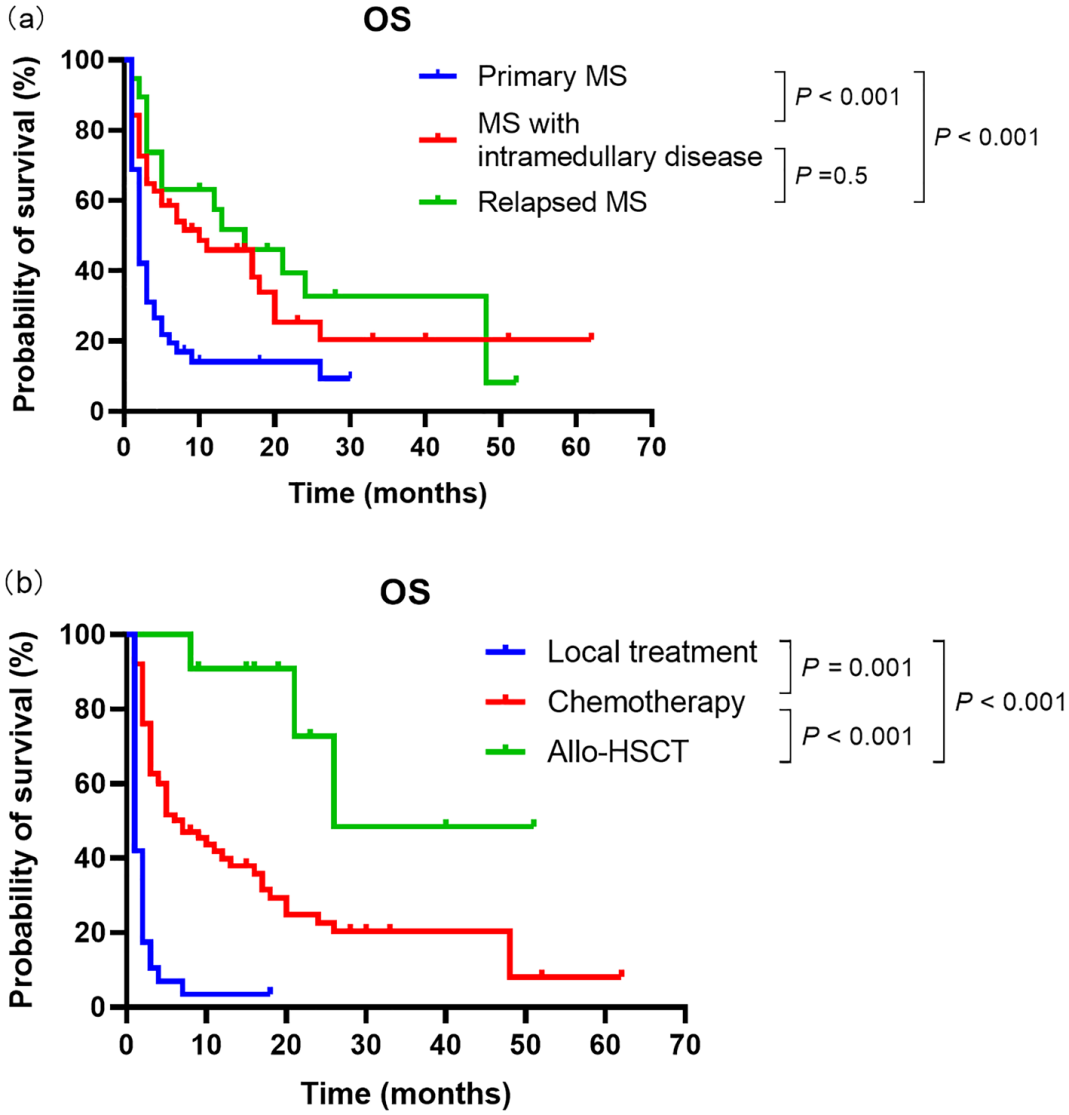
(**a**) Overall survival (OS) according to the myeloid sarcoma subtype: primary myeloid sarcoma (MS, n = 48), myeloid sarcoma with intramedullary disease (n = 51) and relapsed myeloid sarcoma (n = 19). (**b**) OS according to therapy: local treatment (n = 31), chemotherapy (n = 76), and allogenic hematopoietic stem cell transplantation (allo-HSCT, n = 11).

The survival curves of patients with primary myeloid sarcoma (n = 48), myeloid sarcoma with intramedullary disease (n = 51), and relapsed myeloid sarcoma (n = 19) are shown in Fig. 2a. The median survival times of patients in the three groups were 2, 10, and 16 months, respectively. According to the log-rank test, OS differences among the three groups were statistically significant (*P* < 0.001). The survival curves of the patients in the local treatment group (n = 31), the chemotherapy group (n = 76), and the allo-HSCT group (n = 11) are shown in Fig. 2b. The median survival of the patients in the three groups was 1, 7, and 19 months, respectively, with statistically significant differences (*P* < 0.001).

In the total cohort, variables considered to have the potential prognostic value on outcome were myeloid sarcoma subtype (primary/intramedullary/relapsed myeloid sarcoma) and treatment (local treatment/chemotherapy/HSCT). In the multivariate analysis (Table 5), transplantation was an independent protective factor for survival (*P* < 0.001).

**Table 5:** Univariate and multivariate analyses of the 118 patients with MS.

Variables	Univariate analysis			Multivariate analysis		
	HR	95% CI	*P*	HR	95% CI	*P*
Sex (male/female)	1.035	0.672–1.594	0.867	0.774	0.483–1.239	0.285
Age at diagnosis	1.299	0.851–1.983	0.191	1.149	0.729–1.811	0.550
MS subtype	0.586	0.428–0.803	< 0.001	1.514	0.745–3.075	0.518
Sites (≥ 2 sites/single site)	1.346	0.730–2.480	0.304	1.630	0.831–3.195	0.155
Ki-67 index	0.822	0.529–1.279	0.347	0.900	0.565–1.435	0.659
**Treatment**			< 0.001			< 0.001
Local treatment/HSCT	10.448	3.887–28.088	< 0.001	9.378	3.190–27.566	< 0.001
Chemotherapy/HSCT	2.600	1.037–6.520	0.042	2.687	1.030–7.011	0.043

*MS* myeloid sarcoma, *HSCT* hematopoietic stem cell transplantation, *HR* hazard ratio, *CI* confidence interval.

## Discussion

Myeloid sarcoma has various clinical manifestations that may affect many parts of the body. Commonly affected sites, as reported in the literature, include lymph nodes, soft tissue, testis, skin, and mammary gland^12^. Interestingly, the incidence of spinal canal myeloid sarcoma was second only to that of lymph nodes and soft tissue in this study, which reported 15 cases of spinal canal involvement, 8 cases of primary myeloid sarcoma, 6 cases of myeloid sarcoma with intramedullary disease, and 1 case of relapsed myeloid sarcoma. Spinal canal myeloid sarcoma is a rare disease with rapid progression and high mortality^13,14^. In this study, most patients who had myeloid sarcoma with acute spinal cord compression underwent decompression surgery to remove the focus and had pathological samples available to confirm the diagnosis. Of the 15 patients with spinal canal myeloid sarcoma, 6 received only local treatment (surgical treatment), and 5 died within 1 month after diagnosis. Eight patients underwent surgery combined with chemotherapy, and 5 died; the median survival time was 9.5 months. The prognosis was better than that associated with local treatment. One patient underwent HSCT and received maintenance therapy with decitabine after transplantation; this patient has now survived for 9 months after diagnosis.

Cutaneous myeloid sarcoma is characterized by multiple erythematous papules and nodules, most frequently associated with monocyte-containing AML^15^; this form has a high incidence, a poor prognosis, and a short survival time in children^16^. This study included 7 patients with cutaneous myeloid sarcoma, 5 with relapsed myeloid sarcoma, 1 with myeloid sarcoma with intramedullary disease (AML derived from MDS transformation), and 1 with primary myeloid sarcoma. Among these, 6 patients received chemotherapy, but at the end of follow-up, all 6 patients had died. One patient received chemotherapy combined with HSCT and low-dose decitabine maintenance treatment after transplantation. That patient has been observed for 19 months after diagnosis and is in a state of recurrence-free survival.

The t(8;21) translocation is the most frequently reported cytogenetic abnormality associated with myeloid sarcoma and is mainly associated with orbital myeloid sarcoma in children^17^. The inv(16) abnormality is the next most frequent and is associated with abdominal myeloid sarcoma^18^. In this study, only one patient, a 10-year-old child, had orbital myeloid sarcoma with the t(8;21) translocation, and one patient was inv(16) positive for myeloid sarcoma with AML in the digestive tract. Bone marrow samples from patients with myeloid sarcoma at diagnosis were retrospectively analyzed to explore the mutational status of myeloid sarcoma. *KIT* was the most common mutation, followed by *TET2*, *NRAS*, *FLT3-ITD*, *NPM1*, and *DNMT3A*. Eleven (26.8%) of 41 patients with myeloid sarcoma had at least two gene mutations. *KIT* is a common mutation in t(8;21) AML and is most often located at exon 17. The major type of mutation is D816, followed by N822K. The former has the characteristics of high white blood cell count, elevated number of AML1-ETO fusion gene transcripts, and a high recurrence rate^19,20^.

The *KIT* mutation was found in 7 of 10 patients with t(8;21) myeloid sarcoma, and the mutation rate of exon 17D816 was 57%, which was consistent with previous reports. Tyrosine kinase inhibitors (TKIs) can target the *KIT* mutation and KIT protein overexpression, thus improving the remission rate and reducing recurrence. In this study, one patient was treated with a TKI combined with HSCT, and one patient was treated with a TKI combined with chemotherapy. At the end of follow-up, all patients were disease-free and OS was 51 months and 17 months, respectively. In recent years, the *TET2* mutation has been found to play an important role in the pathogenesis of myeloid sarcoma^21^; Furthermore, animal experiments and clinical observations have suggested that decitabine is safe and effective in treating patients with myeloid sarcoma with the *TET2* mutation. Thus, cytogenetics and molecular examination are beneficial for risk stratification and identifying the optimal treatment strategy in patients with myeloid sarcoma.

Primary myeloid sarcoma usually progresses to AML between 5 and 12 months after diagnosis. Surgical resection or radiation therapy can relieve local compression symptoms but cannot delay the progression of the disease. Myeloid sarcoma with intramedullary disease and relapsed myeloid sarcoma has poor prognoses and short survival times. HSCT can be used as first-line therapy for consolidation after induced remission and as salvage therapy for patients with relapsed or refractory disease^3^. In recent years, with the rapid development of cytogenetics and molecular biology, hypomethylating agents (HMA), targeted drugs, and immunotherapy have been shown to be effective.

Studies in both adults and children have shown that HSCT significantly prolongs survival and improves adverse outcomes in myeloid sarcoma^22,23^. The median survival time of 11 patients in this study who underwent HSCT was 19 months, which was significantly better than that of the local treatment group and the chemotherapy group. Frietsch et al.^24^ reported findings from 19 patients with relapsed myeloid sarcoma who underwent allo-HSCT; most patients (16/19) received RIC, suggesting that RIC may increase the risk of extramedullary recurrence after transplantation. There is no uniform standard for the selection of conditioning regimens; most centers choose individualized conditioning regimens according to the patient’s physical condition, disease state, complications, and other factors. Furthermore, the effects of transplant timing, transplant mode, and donor selection on the prognosis of myeloid sarcoma are still under study. Bourlon et al.^25^ retrospectively analyzed 303 patients with AML who underwent allo-HSCT during their first complete remission (CR1), and 39 of them were diagnosed with extramedullary infiltration. There was no difference in OS between the two groups after 3 years of follow-up, suggesting that allo-HSCT performed in the CR1 phase may improve the poor prognosis of AML with extramedullary infiltration.

Most of the current studies involve either matched sibling donors or matched unrelated donors for allogeneic transplantation. Haploidentical HSCT (haplo-HSCT) can be considered in patients without a fully matched HLA donor. Yu et al.^26^ analyzed 14 patients with myeloid sarcoma who received haplo-HSCT and reported 1-year and 3-year survival rates of 71.4% and 64.3%, respectively; the transplant-related mortality was 7%, suggesting that haplo-HSCT is safe and effective in the treatment of myeloid sarcoma. Transplant-related mortality and GVHD may result from allo-HSCT, therefore autologous HSCT has been proposed as an approach to achieve long-term survival in young, chemotherapy-sensitive, and minimal residual disease-negative patients with myeloid sarcoma^27,28^. However, most of the data on autologous HSCT have been derived from case reports.

Recurrence remains the main cause of transplant failure. The reported recurrence rate of myeloid sarcoma after transplantation is 50%^4^. Of the 11 patients in this study who received myeloablative allo-HSCT at the CR1 stage, four patients (36.3%) experienced relapse, and the median time to recurrence was 8 months (range, 6–12 months) after transplantation. Effective prevention of recurrence is the key to improving the efficacy of transplantation. Methods such as immunosuppression reduction, intensive chemotherapy, donor lymphocyte infusion, and secondary transplantation have limited efficacy and an increased incidence of adverse reactions. In recent years, HMAs, molecularly targeted drugs, and cellular immunotherapy have gradually shown good efficacy in preventing relapse.

The antileukemia activity of HMAs differs from that of traditional cytotoxic drugs, and its advantage is the immunomodulatory effects^29^. HMAs significantly enhance the immunogenicity of leukemic cells; conversely, they regulate the function of immune cell subsets such as NK cells and CD8^+^ T cells to enhance the killing effect. Several reports have shown that HMAs, through these mechanisms, can be used alone in elderly patients with myeloid sarcoma^30^ or combination with chemotherapy^31^, radiotherapy^32^, and targeted therapy^33^ to increase the antitumor effect. In patients with the *TET2* mutation, decitabine may be a useful treatment option^21^.

Downregulation of HLA-II expression caused by hypermethylation of promoter class II transactivator is a mechanism responsible for the immune escape of leukemic cells after transplantation for AML^34,35^. HMAs play a role in GVL reactions by upregulating the expression of silenced WT1 tumor antigen and HLA-II molecules through demethylation. Decitabine and azacytidine have also been safely used in post-transplant maintenance therapy for AML, MDS, and acute lymphoblastic leukemia^9–11,36^. HMAs are equally effective and safe in the treatment of extramedullary recurrent myeloid sarcoma after transplantation^37–39^. Our center attempted treatment with low-dose decitabine (10 mg/d for 3 days every 4 weeks for eight cycles) after transplantation to prevent recurrence. In this study, 11 patients received allo-HSCT, of which six patients received maintenance therapy with decitabine after transplantation. Of the 6 patients, 2 harbored a *TET2* mutation. Five patients survived at the end of follow-up, and no recurrence was found at 9, 19, 23, 40, and 51 months after diagnosis.

Kawamoto et al.^2^ analyzed 131 patients with myeloid sarcoma and found that myeloid sarcoma secondary to MDS/MPN/CML had a poor prognosis, which may have been related to chemotherapy resistance caused by previous treatment. Some success has been reported in patients with refractory/recurrent myeloid sarcoma. Heudobler et al.^40^ reported findings from a patient with high-risk MDS and skin myeloid sarcoma: monotherapy with HMA was ineffective, but treatment combined with a biomodulatory approach resulted in complete remission of skin myeloid sarcoma. Kanate et al.^41^ reported findings from a patient with refractory myeloid sarcoma who achieved complete remission with single-agent venetoclax. Kida et al.^42^ reported the results of HSCT in a patient who had extramedullary relapse myeloid sarcoma with *FLT3-ITD* mutation and resistance to chemotherapy; monotherapy with gilteritinib (120 mg/d) resulted in complete remission. Furthermore, cytotoxic T lymphocyte–associated protein 4 inhibitors^43^ and CD33 monoclonal antibodies^44^ have successfully treated myeloid sarcoma.

In summary, myeloid sarcoma is a rare disease in the clinic. Our study analyzed the clinical features, treatment, and prognosis of 118 patients with myeloid sarcoma. We found HSCT can significantly improve the prognosis of myeloid sarcoma and recurrence is the main reason for treatment failure after transplantation. Maintenance therapy with decitabine after myeloid sarcoma transplantation may prolong the survival of patients, but this finding must be confirmed by large-sample clinical trials.

## Methods

### Patients

We retrospectively analyzed 118 patients who were diagnosed with myeloid sarcoma according to the WHO criteria by at least 2 expert hematopathologists at the First Affiliated Hospital of Zhengzhou University from 1 January 2010 to 1 July 2021.

### Diagnostic criteria and methods

Myeloid sarcoma, as a specific type of AML, is defined by the WHO as a mass formed by the accumulation of myeloid blasts in anatomical sites other than the bone marrow, and its diagnosis is based on the disruption of normal tissue structure. For this reason, the patients included in this study were all myeloid sarcoma patients confirmed by histopathological biopsy, and the central nervous system leukemia only manifested as blast cells found in the cerebrospinal fluid test was excluded.

All specimens were fixed in 10% neutral formalin, embedded in paraffin, sectioned and stained with HE and observed under a microscope. The morphology of myeloid sarcoma shows infiltration of myeloid cells in different stages. Cells were medium-sized with a relatively homogeneous morphology, large nuclei, round or ovoid, and little cytoplasm. Immunohistochemistry is an essential method for the diagnosis of myeloid sarcoma. When tumor cells express at least one myeloid marker (MPO, CD13, or CD33) but not express T cell markers (CD3, CD5, or CD7), myeloid sarcoma can be diagnosed. In addition, immunohistochemical tests for TCRαβ and TCRγδ are performed on patients with positive T-cell markers, and if both TCRαβ and TCRγδ are negative, myeloid sarcoma with T-cell markers is diagnosed. Each marker was considered positive when expressed by at least 20% of tumor cells.

After the diagnosis of myeloid sarcoma was confirmed by pathology, a bone marrow biopsy was performed to determine whether there was concomitant bone marrow infiltration, and the immunophenotype was detected by flow cytometry; the karyotype was analyzed by G-banding method, and the myeloid mutations were detected by next-generation sequencing. Bone marrow was reviewed every 1–3 months during treatment. We routinely detected MRD by flow cytometry and QT-PCR. Evaluation of extramedullary lesions using ultrasound, CT, MRI, or PET/CT.

### MS subgroups

We retrospectively analyzed 118 patients with myeloid sarcoma and divided them into three groups: myeloid sarcoma without peripheral blood and bone marrow involvement (primary myeloid sarcoma), myeloid sarcoma with intramedullary disease (including AML, MDS, MPN, and CML), and relapsed myeloid sarcoma after remission of AML. Data regarding the age, sex, involved site, disease type, chromosome karyotype analysis, mutant gene, immunohistochemistry, treatment, follow-up, and outcomes were collected.

### Treatment

According to different treatment methods, 118 patients with myeloid sarcoma were divided into three groups. The first group was the local treatment group, which included 31 patients (26.2%) who received local radiotherapy, surgical treatment, and intrathecal injection. The second group was the chemotherapy group; 76 patients (64.4%) underwent a chemotherapy regimen that contained cytarabine and anthracycline, and the third group involved allo-HSCT in 11 patients (9.4%). Among the allo-HSCT group, 8 patients underwent human leukocyte antigen (HLA) matched sibling donor transplantation, 2 underwent HLA matched unrelated donor transplantation, and 1 underwent haploidentical donor transplantation.

### Statistical analysis

OS was defined as the time from the date of diagnosis to death from any cause or the last follow-up. Statistical analyses were performed with SPSS software (version 25.0) and GraphPad Prism (version 8.0). The Kaplan–Meier method was used to analyze the univariate survival according to age, sex, single or multiple involved sites, Ki-67 index, disease type, and treatment method. The log-rank test was used to compare the significant differences between groups. COX multivariate analysis was used to analyze the independent factors affecting the survival of MS patients. All comparisons were analyzed by bilateral tests and *P* < 0.05 was considered statistically significant.

### Ethics approval and consent to participate

Informed consent was obtained from all patients or legal guardians and the study protocol was approved by the Ethics Committee of the First Affiliated Hospital of Zhengzhou University (Number SS-2018-42) and based on the ethical principles for medical research involving human subjects of the Helsinki Declaration.

### Consent for publication

All authors agreed to publish.

## Data Availability

Data and material will be available upon corresponding author approval. All data sets generated/analyzed for this study are included in the manuscript and the additional files.
